# Opposing associations of depression with sexual behaviour: implications for epidemiological investigation among gay, bisexual and other men who have sex with men

**DOI:** 10.1136/sextrans-2020-054634

**Published:** 2021-01-11

**Authors:** Ada R Miltz, Alison J Rodger, Andrew N Phillips, Janey Sewell, Simon Edwards, Sris Allan, Lorraine Sherr, Anne M Johnson, William J Burman, Fiona C Lampe, ASTRA and AURAH Study Groups, Andrew Speakman

**Affiliations:** 1 Institute for Global Health, University College London, London, UK; 2 Mortimer Market Centre, Central and North West London NHS Foundation Trust, London, UK; 3 City of Coventry NHS Healthcare Centre, Coventry, UK; 4 Denver Public Health, Denver, Colorado, USA

**Keywords:** epidemiology (general), gay men, HIV, sexual behaviour

## Abstract

**Objective:**

The aim of this report is to investigate the nature of the relationship between depression and condomless sex (CLS) among gay, bisexual and other men who have sex with men (GBMSM).

**Methods:**

Data are from the Antiretrovirals, Sexual Transmission Risk and Attitude (ASTRA) study of people living with HIV and attending one of eight HIV outpatient clinics in England (2011–2012) and the Attitudes to and Understanding of Risk of Acquisition of HIV (AURAH) study of HIV-negative/unknown status individuals attending one of 20 genitourinary medicine clinics in England (2013–2014). This analysis included GBMSM only. For each study, the prevalence of depressive symptoms (Patient Health Questionnaire-9 score ≥10) was presented according to three categories of sex in the past 3 months (considering anal/vaginal sex with men/women and anal sex with men in separate definitions): (1) no sex, (2) condom-protected sex only and (3) CLS. Multinomial logistic regression with ‘condom-protected sex only’ as the reference group was used to adjust for age and (for ASTRA participants) time since HIV diagnosis.

**Results:**

There were opposing associations of depression with recent sexual behaviour: the prevalence of depression was higher among those who reported no sex and those who reported CLS, compared with those who reported condom-protected sex only. Among the 2170 HIV-positive GBMSM in ASTRA, considering anal/vaginal sex with men/women, the prevalence of depressive symptoms was 32%, 20% and 28%, respectively, among men reporting no sex (n=783), condom-protected sex only (n=551) and CLS (n=836) (global p<0.001). Among the 1477 HIV-negative GBMSM in AURAH, the prevalence of depressive symptoms was 12%, 8% and 13%, respectively, for no sex (n=137), condom-protected sex only (n=487) and CLS (n=853) (global p=0.017). Patterns were similar after adjustment and when only considering anal sex between men.

**Conclusions:**

Depression may be linked both to lack of sexual activity and to sexual risk taking. When investigating associations between depression and CLS, it is important to separate out individuals reporting condom-protected sex only from those reporting no sex.

## Introduction

A meta-analysis carried out in 1999 concluded there was little evidence for an association between depressive symptoms and sexual behaviour linked to STI/HIV transmission risk among gay, bisexual and other men who have sex with men (GBMSM) as findings across studies were inconsistent.^1^ A number of studies of GBMSM in high-income countries^2–20^ (sample size ranging from 120^2^ to 4295^17^) have since found moderate to strong associations between depression and measures of condomless sex (CLS) even after controlling for sociodemographic and psychosocial factors. Others have found no such association in unadjusted or adjusted analysis,^21–26^ although many have been limited by lack of power.

Theories have been put forth to explain the inconsistency of findings on the association between depression and sexual risk taking.^1^ For instance, it has been hypothesised that there is a non-linear relationship between depression severity and sexual risk behaviour whereby the presence of moderate depressive symptoms may be associated with increased sexual risk behaviour, and severe symptoms may have the opposite effect by reducing sexual interest and libido. However, in a number of studies that investigated this hypothesis, the highest level of depression severity was associated with increased reporting of CLS measures compared with lower but still clinically significant levels of depression.^6 8 9 14 16 17 19^ Another theory suggests that there are opposing associations of depressive symptoms: depression may result in increased sexual risk behaviour in some individuals, and in others sexual inactivity due to lowered libido and loss of interest in sex, or reductions in self-confidence and self-esteem.^1^ The type of coping mechanism employed may possibly determine the direction of association. Some people may cope with depression with externalising behaviours such as sexual risk taking and substance use, and others with internalising behaviours including social withdrawal and sexual inactivity.^w1^ It has been suggested in genetic studies that the presence of certain genes (including the serotonin transporter gene, serotonin 2A receptor gene and dopamine D4 receptor gene) may confer risk for the co-occurrence of depression and externalising disorders such as substance use.^w1–w8^ Environmental factors may also confer risk for the co-occurrence of depression and externalising disorders.^w1^ In addition, some antidepressants may impact psychosexual functioning in some individuals.^w9^

Under the hypothesis of opposing associations, the prevalence of depression would be elevated both among men who report lack of sexual activity and among men who report sex linked to risk of STI/HIV transmission (eg, CLS) and would be lower among men who report ‘safe sex’ (eg, condom-protected sex). Therefore, in a study that combines participants who report no recent sexual intercourse with those who report condom-protected sex into a single ‘no CLS’ comparator group, the association between depression and CLS may be diluted or obscured. In existing studies, sexually inactive men were not always actively separated or excluded from the ‘safe sex’ comparator group. It is possible that the relative frequency of these participants may contribute to the inconsistency of findings on depression in STI/HIV prevention research.

The aim of this report is to investigate the hypothesis of opposing associations within samples of HIV-positive GBMSM and HIV-negative GBMSM, by assessing the association of depressive symptoms with recent CLS, considering ‘no sex’ and ‘condom-protected sex’ separately. Data are from the Antiretrovirals, Sexual Transmission Risk and Attitudes (ASTRA) study of people living with HIV (2011–2012) and the Attitudes to and Understanding of Risk of Acquisition of HIV (AURAH) study of HIV-negative or of unknown status individuals (2013–2014).

## Methods

In this paper, data were analysed from two separate cross-sectional questionnaire studies in England. The ASTRA study recruited HIV-positive men and women aged 18 years or older from one of eight HIV outpatient clinics in England between February 2011 and December 2012. The AURAH study recruited HIV-negative or unknown HIV status men and women aged 18 years or over from one of 20 genitourinary medicine (GUM) clinics in England between June 2013 and November 2014. Methodological details of both studies have been published elsewhere.^27 28^

### GBMSM included in analysis

Men were classified as GBMSM if they reported being gay or bisexual (including other plurisexual identity labels) or reported anal sex with a man in the past 3 months. In AURAH, men who reported having disclosed to their family, friends or workmates as being gay, bisexual and/or attracted to men were also classified as GBMSM. This question was not asked in ASTRA. HIV-positive GBMSM in ASTRA were excluded from analysis if they were diagnosed with HIV less than 3 months ago, given that the recall period for sexual behaviour was the past 3 months. Four GBMSM in the AURAH study tested positive for HIV on the day of recruitment. These HIV-positive men were retained in the sample for analyses, as they were not diagnosed with HIV at the time of questionnaire completion. The AURAH sample is hereafter referred to as ‘HIV-negative’.

### Depressive symptoms

In both studies, the Patient Health Questionnaire (PHQ-9) was used to assess depressive symptoms, which enquires whether the following symptoms have occurred in the previous 2 weeks: anhedonia, feeling down, sleeping problems, fatigue, appetite disturbance, feeling like a failure, difficulties with concentration, psychomotor retardation and thoughts of suicide/self-harm. Each symptom occurrence is rated from 0 (not at all) to 3 (nearly every day); the total score ranges from 0 to 27. A missing response was considered to indicate that the symptom had not occurred, as there appeared to be a common response pattern in which only those symptoms that had occurred were ticked on the questionnaire. A total score of 10 or greater on PHQ-9 was considered to indicate a possible clinically significant depressive condition.^18^ The score was further categorised as follows: moderate (score 10–14), moderately severe (score 15–19) and severe symptoms (score 20–27) of depression.

### Sexual behaviour

Two classifications of sexual behaviour in the past 3 months were used. The first was based on anal sex with men or vaginal or anal sex with women, given the inclusion of bisexual identified men. The second was based only on anal sex with men. For each, sexual behaviour in the past 3 months was classified into one of the following mutually exclusive groups: (1) no sex, (2) condom-protected sex only and (3) CLS. Individuals for whom there was insufficient information to categorise sexual behaviour were excluded from analysis (<5% in both studies).

In this study, CLS was used as a measure of sexual behaviour linked to STI/HIV acquisition and/or transmission risk among HIV-negative and HIV-positive GBMSM. It is important to acknowledge that the act of CLS will often occur within the context of loving and mutually supportive relationships. In an additional analysis, CLS with one long-term partner only is grouped together with ‘condom-protected sex only’. It is also important to note that in terms of HIV transmission specifically, alternative risk reduction strategies, other than condom use, are also relevant.^w10 w11^

### Statistical analysis

Considering ASTRA and AURAH separately, the prevalence of depressive symptoms according to sexual behaviour category was assessed. Multinomial logistic regression was used to investigate the relationship between depressive symptoms and categories of sexual behaviour: no sex versus condom-protected sex and CLS versus condom-protected sex. Odds ratios (OR) are presented unadjusted and adjusted for age group (18–29, 30–39, 40–49, 50+ years), and for ASTRA participants only, additionally adjusted for time since HIV diagnosis (3 months-2 years, 2–5 years, 5–10 years, 10–15 years, 15–20 years, ≥20 years). For age and time since HIV diagnosis (in ASTRA), the proportion of missing responses was <5% in both studies.

Logistic regression was then used to assess the impact of including sexually inactive GBMSM in the ‘no CLS’ comparator group when investigating the association between depressive symptoms (score ≥10 and categories of severity) and anal/vaginal CLS.

Finally, as CLS with one long-term partner only might not be considered a significant risk for STI/HIV transmission, an additional analysis was carried out for both studies whereby the condom-protected sex category was expanded to include this group, considering anal/vaginal sex.

All analyses were performed in STATA V.15 statistical software^29^ and reported according to the guidelines of the Strengthening the Reporting of Observational Studies in Epidemiology.

## Results

### Characteristics of HIV-positive GBMSM in the ASTRA study

In total, 2170 HIV-positive GBMSM in ASTRA who had been diagnosed with HIV for at least 3 months and who had complete data were included. Overall, 95.4% identified as gay and 4.6% as bisexual or another plurisexual identity label. Ninety per cent of men were of white ethnicity; the median age was 46 years (IQR 40–52). Overall, 70.1% were born in the UK, 43.7% had a university degree and 67.7% were attending a clinic in London. Time since HIV diagnosis was 3 months-2 years, 2–5 years, 5–10 years, 10–15 years, 15–20 years and ≥20 years for 8.5%, 15.6%, 25.2%, 21.2%, 17.3% and 12.3% of men, respectively. The prevalence of depressive symptoms (PHQ-9 ≥10) was 27.4% (n=594); moderate, moderately severe and severe symptoms were reported by 13.5%, 7.8% and 6.0%, respectively. The three categories of anal/vaginal sex (no sex, condom-protected sex only, CLS) were reported by 783 (36.1%), 551 (25.4%) and 836 (38.5%) men, respectively.

### Depression and sexual behaviour among HIV-positive GBMSM in the ASTRA study

**Figure 1:**
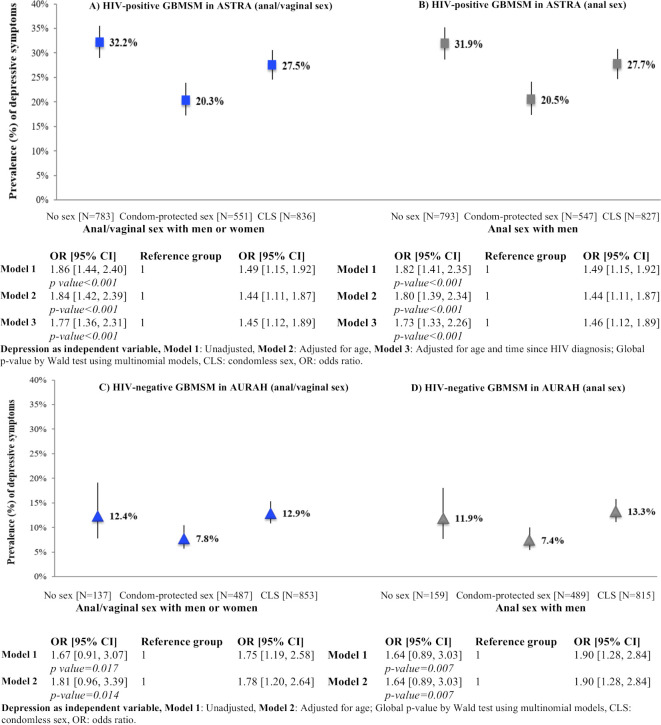
Prevalence of depression (PHQ-9 ≥10) according to sexual behaviour (past 3 months) among HIV-positive GBMSM in ASTRA and HIV-negative GBMSM in AURAH (scale is to 40%, lines represent 95% CI). The part labels A, B, C, D indicate the study sample and classification of sexual behaviour investigated. ASTRA, Antiretrovirals, Sexual Transmission Risk and Attitudes; AURAH, Attitudes to and Understanding of Risk of Acquisition of HIV; GBMSM, gay, bisexual and other men who have sex with men; PHQ-9, Patient Health Questionnaire 9.

There was evidence for opposing associations of depression with sexual behaviour among HIV-positive GBMSM in ASTRA. Considering anal/vaginal sex, the prevalence of depressive symptoms was higher among men who reported no sex (32.2%) and those who reported CLS (27.5%), compared with those who reported condom-protected sex only (20.3%) (figure 1A). A very similar pattern was apparent when considering depression prevalence by categories of anal sex between men: 31.9% for no anal sex and 27.7% for anal CLS, compared with 20.5% for condom-protected anal sex only (figure 1B). The pattern of associations was similar after adjustment for age and time since HIV diagnosis (figure 1).

When including only the 1387 sexually active GBMSM in ASTRA, the OR for the association between depressive symptoms (PHQ-9 ≥10) and anal/vaginal CLS was 1.49 (95% CI 1.15 to 1.92) (p=0.002), and the ORs for moderate, moderately severe and severe depressive symptoms (vs PHQ-9 <10) were 1.31 (95% CI 0.94 to 1.82), 1.45 (95% CI 0.94 to 2.23) and 2.38 (95% CI 1.29 to 4.38), respectively (global p=0.009). When all 2170 GBMSM in ASTRA were included, no association between depression and anal/vaginal CLS was apparent: 1.01 (95% CI 0.83 to 1.23) (p=0.909) for PHQ-9 ≥10 (vs <10), and 1.05 (95% CI 0.81 to 1.35), 1.07 (95% CI 0.77 to 1.47) and 0.87 (95% CI 0.60 to 1.26) for moderate, moderately severe and severe depressive symptoms (vs PHQ-9 <10), respectively (global p=0.815).

The pattern of depression prevalence was similar when adding CLS with one long-term partner only to the ‘condom-protected sex’ category in ASTRA. Considering anal/vaginal sex, the prevalence of depressive symptoms was 32.2% for no sex (n=783) and 30.0% for CLS with at least one partner who was not a long-term partner (n=530), compared with 20.9% for condom-protected sex only or CLS with one long-term partner only (n=819).

### Characteristics of HIV-negative GBMSM in the AURAH study

In total, 1477 HIV-negative GBMSM in AURAH with complete data were included in the current analysis. Overall, 88.9% identified as gay, 9.5% as bisexual or another plurisexual identity label and 1.6% as straight. Eighty-two per cent of men were of white ethnicity; the median age was 32 years (IQR 26–40). Overall, 58.0% were born in the UK, 66.9% had a university degree and 75.0% were attending a clinic in London. The prevalence of depressive symptoms (PHQ-9 ≥10) was 11.2% (n=165); 6.8%, 3.1% and 1.3% of men reported moderate, moderately severe and severe symptoms of depression, respectively. The three categories of anal/vaginal sex (no sex, condom-protected sex only, CLS) were reported by 137 (9.3%), 487 (33.0%) and 853 (57.8%) men, respectively.

### Depression and sexual behaviour among HIV-negative GBMSM in the AURAH study

There was also evidence for opposing associations of depression with sexual behaviour among HIV-negative GBMSM in AURAH. Considering anal/vaginal sex, the prevalence of depressive symptoms was higher among men who reported no sex (12.4%) and among men who reported CLS (12.9%) compared with men who reported condom-protected sex only (7.8%) (figure 1C). Similarly, when only considering anal sex between men, the prevalence of depressive symptoms was 11.9% for no sex and 13.3% for CLS, compared with 7.4% for condom-protected sex only (figure 1D). The pattern of associations was similar after adjustment for age (figure 1).

When including only the 1340 sexually active GBMSM in AURAH, the OR for the association between depression (PHQ-9 ≥10) and anal/vaginal CLS was 1.75 (95% CI 1.19 to 2.58) (p=0.005), and the ORs for moderate, moderately severe and severe depressive symptoms (vs PHQ-9 <10) were 1.34 (95% CI 0.84 to 2.12), 3.02 (95% CI 1.33 to 6.86) and 2.62 (95% CI 0.74 to 9.24), respectively (global p=0.016). When all 1477 GBMSM in AURAH were included, the associations were weaker: 1.53 (95% CI 1.09 to 2.16) for PHQ-9 ≥10 (vs <10) (p=0.014), and 1.25 (95% CI 0.82 to 1.90), 2.44 (95% CI 1.23 to 4.84) and 1.66 (95% CI 0.63 to 4.39) for the respective severity categories (global p=0.041).

The pattern of depression prevalence was similar when adding CLS with one long-term partner only to the ‘condom-protected sex’ category in AURAH. Considering anal/vaginal sex, the prevalence of depressive symptoms was 12.4% for no sex (n=137) and 14.5% for CLS with at least one partner who was not a long-term partner (n=616), compared with 7.8% for condom-protected sex only or CLS with one long-term partner only (n=714).

## Discussion

Findings in this study of HIV-positive and HIV-negative GBMSM provide evidence in support of the theory of opposing associations of depression with sexual behaviour. Considering CLS as the behaviour linked to STI/HIV transmission and studying ‘no sex’ and ‘condom-protected sex’ separately produced opposing associations whereby the prevalence of depressive symptoms was higher among sexually inactive men and men who reported CLS than among men who reported condom-protected sex only. The same pattern was apparent among HIV-positive and HIV-negative GBMSM, although the prevalence of depressive symptoms was consistently higher in the former sample. The associations remained after adjustment for age and, for HIV-positive men, time since HIV diagnosis. These findings were observed regardless of whether the definition of sexual behaviour included anal or vaginal sex with women, or was restricted to anal sex with men. Adding men who reported CLS with one long-term partner only to the ‘condom-protected sex’ group produced the same pattern of association.

For both HIV-positive and HIV-negative GBMSM, an analysis of the whole sample, in which men who reported no sex were grouped together with those who reported condom-protected sex only into a ‘no CLS’ comparator group, resulted in a dilution of the relationship between depressive symptoms and CLS. This relationship was fully attenuated in ASTRA and partially attenuated in AURAH, since the AURAH sample included a lower proportion of sexually inactive men than the ASTRA sample (9% vs 36%). The relative frequency of sexually inactive men may contribute to the inconsistency of findings across studies investigating the association of depression with CLS among GBMSM. We identified a subset of 11 of these studies that stated the proportion of participants reporting recent anal sex with a man, either in the results or implicit as part of the inclusion criteria. In 9 out of these 11 studies, all or almost all participants (98%–100%) reported recent sex.^2 6–8 11 12 14 16 17 19 22^ Eight of these nine studies found a significant and positive association of depressive symptoms with measures of CLS. In the other two studies, the prevalence of recent anal sex with a man was lower, 60% (past 3 months)^24^ and 72% (past year),^26^ and neither found evidence of an association in unadjusted or adjusted analysis. This provides some further support to the hypothesis that including sexually inactive men in the ‘safe sex’ comparator group can dilute the association between depressive symptoms and CLS due to opposing associations of depression with sexual behaviour.

These findings have important implications for epidemiological investigation in STI/HIV prevention research. It is recommended that when assessing the association between depression and sexual risk taking, rather than comparing ‘sexual risk behaviour’ with ‘no sexual risk behaviour’, future studies should separate out individuals reporting ‘safe sex’ from those reporting no sex. This is in order to fully investigate the nature of the relationship and ensure that opposing associations do not cancel each other out. Such consideration of opposing associations may facilitate further exploration of the extent to which the relationship between depressive symptoms and CLS is likely to be causal, and the possible mechanisms of effect. In a previous analysis of sexually active GBMSM in AURAH, using structural equation modelling, there was evidence that depression was associated with CLS either by lowering one’s self-efficacy for sexual safety or by leading to higher levels of recreational drug use, on a distinct causal pathway.^30^ Similar findings have been observed in previous studies of sexually active US GBMSM^11^ and South African GBMSM.^w12^ Data are needed to better understand the role of other factors such as intimate partner violence on the causal pathway between depression and STI/HIV transmission risk. It is also important that future studies address the ‘opposite’ role of depression in lowered libido and sexual inactivity among GBMSM, and investigate the complex role that antidepressants may play in this relationship. A better understanding of the mechanisms by which depression impacts on sexual behaviour could help guide interventions. It is also important to note that any association between depression and sexual behaviour may be bidirectional: specific patterns of sexual behaviour may impact on mental health, making the symptoms worse, prolonging episodes or even triggering the onset of depression.

In this study, CLS was considered the most appropriate measure to capture STI/HIV transmission risk among HIV-negative and HIV-positive GBMSM. Studies specifically concerned with HIV transmission risk only would need to incorporate information on HIV status of partners, and additional factors now known to protect against HIV transmission, in particular HIV viral suppression on antiretroviral therapy and use of pre-exposure prophylaxis (PrEP).^w10 w11^ Evidence related to these additional factors was limited at the time of the AURAH and ASTRA studies, and the prevalence of PrEP use was very low. It is possible that opposing associations of depression may still apply in the context of widespread PrEP use. There is some evidence that GBMSM who use PrEP may experience lower levels of generalised anxiety than those engaging in CLS who do not use PrEP.^w13^ Studies investigating the association between depression and PrEP use/adherence and potential mechanisms related to this are needed.

AURAH recruited from GUM clinics and as such sexually inactive participants may represent a distinct subset of men who may not be generalisable to sexually inactive HIV-negative GBMSM in the population in England. Both ASTRA and AURAH were cross-sectional observational studies; causal links between depression and CLS may operate in either or both directions.

Gay and plurisexual identified men may have different needs and experiences. There is a growing body of evidence to suggest that bisexual identified men experience a greater burden of depressive symptomatology than do gay identified men.^w14^ Due to small numbers, it was not possible to investigate gay and plurisexual identified men separately in this analysis.

## Conclusions

In conclusion, depression has a complex relationship with sexual behaviour among GBMSM, potentially being linked both to lack of sexual activity and to sexual risk taking. Inconsistent findings of the relationship between depression and sexual risk behaviour among GBMSM may, in part, be explained by the inclusion of sexually inactive men in the sample.

**Key messages**

- Depression was associated both with increased sexual inactivity and with increased condomless sex (CLS), compared with condom-protected sex, providing evidence in support of the theory of opposing associations of depression with sexual behaviour.
- When adding men who reported no sex to the condom-protected sex comparator group, the relationship between depressive symptoms and CLS was diluted.
- The relative frequency of sexually inactive men may contribute to the inconsistency of findings on depression in STI/HIV prevention research.
- Future studies should separate out individuals reporting ‘safe sex’ from those reporting no sex.

10.1136/sextrans-2020-054634.supp1 Supplementary data

## Data Availability

Data are available upon reasonable request. We have a number of planned analyses for the ASTRA and AURAH studies, but welcome proposals for additional analysis. Please contact Dr Fiona Lampe (f.lampe@ucl.ac.uk). The Study Core Group will review proposals.
