# Supplementary material for: Opposing associations of depression with sexual behaviour: implications for epidemiological investigation among gay, bisexual and other men who have sex with men
Source: Sex Transm Infect. 2021 Jan 11;97(8):613–8. doi: 10.1136/sextrans-2020-054634 (PMC8606445; doi:10.1136/sextrans-2020-054634)
Supplement: Supplementary data [file sextrans-2020-054634supp001.pdf]

Web appendix:

w1. Cosgrove VE, Rhee SH, Gelhorn HL, et al. Structure and etiology of co-occurring internalizing and externalizing disorders in adolescents. *J Abnorm Child Psychol* 2011;39(1):109-23. doi: 10.1007/s10802-010-9444-8 [published Online First: 2010/08/05]

w2. O'Connor TG, McGuire S, Reiss D, et al. Co-occurrence of depressive symptoms and antisocial behavior in adolescence: a common genetic liability. *J Abnorm Psychol* 1998;107(1):27-37. [published Online First: 1998/03/20]

w3. Caspi A, Sugden K, Moffitt TE, et al. Influence of life stress on depression: moderation by a polymorphism in the 5-HTT gene. *Science* 2003;301(5631):386-9. doi: 10.1126/science.1083968 [published Online First: 2003/07/19]

w4. Sakai JT, Young SE, Stallings MC, et al. Case-control and within-family tests for an association between conduct disorder and 5HTTLPR. *Am J Med Genet B Neuropsychiatr Genet* 2006;141B(8):825-32. doi: 10.1002/ajmg.b.30278 [published Online First: 2006/09/15]

w5. Eley TC, Sugden K, Corsico A, et al. Gene-environment interaction analysis of serotonin system markers with adolescent depression. *Mol Psychiatry* 2004;9(10):908-15. doi: 10.1038/sj.mp.4001546 [published Online First: 2004/07/09]

w6. Mik HM, Ehtesham S, Baldassarra L, et al. Serotonin system genes and childhood-onset aggression. *Psychiatr Genet* 2007;17(1):11. doi: 10.1097/YPG.0b013e3280114103 [published Online First: 2006/12/15]

w7. Lopez Leon S, Croes EA, Sayed-Tabatabaei FA, et al. The dopamine D4 receptor gene 48-base-pair-repeat polymorphism and mood disorders: a meta-analysis. *Biol Psychiatry* 2005;57(9):999-1003. doi: 10.1016/j.biopsych.2005.01.030 [published Online First: 2005/04/30]

w8. Rowe DC, Stever C, Chase D, et al. Two dopamine genes related to reports of childhood retrospective inattention and conduct disorder symptoms. *Mol Psychiatry* 2001;6(4):429-33. doi: 10.1038/sj.mp.4000874 [published Online First: 2001/07/10]

w9. Higgins A, Nash M, Lynch AM. Antidepressant-associated sexual dysfunction: impact, effects, and treatment. *Drug Healthc Patient Saf* 2010;2:141-50.

w10. McCormack S, Dunn DT, Desai M, et al. Pre-exposure prophylaxis to prevent the acquisition of HIV-1 infection (PROUD): effectiveness results from the pilot phase of a pragmatic open-label randomised trial. Lancet 2016;387(10013):53-60. doi: 10.1016/s0140-6736(15)00056-2 [published Online First: 2015/09/14]

w11. Rodger AJ, Cambiano V, Bruun T, et al. Risk of HIV transmission through condomless sex in serodifferent gay couples with the HIV-positive partner taking suppressive antiretroviral therapy (PARTNER): final results of a multicentre, prospective, observational study. *Lancet* 2019 doi: 10.1016/S0140-6736(19)30418-0 [published Online First: 2019/05/06]

w12. Tucker A, Liht J, de Swardt G, et al. An exploration into the role of depression and self-efficacy on township men who have sex with men's ability to engage in safer sexual practices. *AIDS Care* 2013;25(10):1227-35. doi: 10.1080/09540121.2013.764383 [published Online First: 2013/02/08]

w13. Keen P, Hammoud MA, Bourne A, et al. Use of HIV Pre-exposure Prophylaxis (PrEP) Associated With Lower HIV Anxiety Among Gay and Bisexual Men in Australia Who Are at High Risk of HIV Infection: Results From the Flux Study. *J Acquir Immune Defic Syndr* 2020;83(2):119-25. doi: 10.1097/qai.0000000000002232 [published Online First: 2020/01/15]

w14. Hickson F, Davey C, Reid D, et al. Mental health inequalities among gay and bisexual men in England, Scotland and Wales: a large community-based cross-sectional survey. *J Public Health (Oxf)* 2017;39(2):266-73. doi: doi:10.1093/pubmed/fdw021
